# Genome of *Tenualosa ilisha* from the river Padma, Bangladesh

**DOI:** 10.1186/s13104-018-4028-8

**Published:** 2018-12-22

**Authors:** Avizit Das, Peter Ianakiev, Abdul Baten, Rifath Nehleen, Tasneem Ehsan, Oly Ahmed, Mohammad Riazul Islam, M. Niamul Naser, Mong Sano Marma, Haseena Khan

**Affiliations:** 1 Department of Biochemistry and Molecular Biology, University of Dhaka, Dhaka, 1000 Bangladesh; 2 Hera Biosciences LLC, Medford, MA USA; 3 AgResearch, Grasslands Research Centre, Palmerston North, 4442 New Zealand; 4 Southern Cross Plant Science, Southern Cross University, Lismore, NSW Australia; 5 Department of Zoology, University of Dhaka, Dhaka, 1000 Bangladesh; 6 Qiagen Sciences, Waltham, MA USA

**Keywords:** Hilsa shad, *Tenualosa ilisha*, Clupediae, Whole genome sequence, NGS platform

## Abstract

**Objective:**

Hilsa shad (*Tenualosa ilisha*), is a popular fish of Bangladesh belonging to the Clupeidae family. An anadromous species, like the salmon and many other migratory fish, it is a unique species that lives in the sea and travels to freshwater rivers for spawning. During its entire life, *Tenualosa ilisha* migrates both from sea to freshwater and vice versa.

**Data description:**

The genome of *Tenualosa ilisha* collected from the river Padma of Rajshahi, Bangladesh has been sequenced and its de novo hybrid assembly and structural annotations are being reported here. Illumina and PacBio sequencing platforms were used for high depth sequencing and the draft genome assembly was found to be 816 MB with N50 size of 188 kb. MAKER gene annotation tool predicted 31,254 gene models. Benchmarking Universal Single-Copy Orthologs refer 95% completeness of the assembled genome.

## Objective

Hilsa shad known as ilish in Bangladeshis popular for its taste and the texture of its flesh. This species of fish belongs to the shad in Clupeidae family. In addition to the Bay of Bengal and riverine Bangladesh (the Padma, Jamuna, Meghna, and other coastal rivers of Bangladesh), this fish is also found in the Persian Gulf, Mediterranean Sea, Arabian Sea and China Sea [1]. Fisheries, a part of the Bangladesh’s cultural heritage, have played an important role on its socioeconomic development in terms of protein supply, generation of employment and earning of foreign currency. According to the FAO, in 2018 Bangladesh ranked 3rd in the world in inland fish production. Hilsa (*Tenualosa ilisha*), is the most popular among the 650 or so marine and inland fish found in Bangladesh. It contributes to 11% of total fish production and 1% to the national GDP, 3.00% of the total export earnings and about 2.5 million people in Bangladesh are directly dependent on Hilsa in providing for their families [2, 3]. At present more than 60% of global Hilsa catch is reported from Bangladesh, 20–25% from Myanmar, 15–20% from India and 5–10% from other countries (e.g., Iraq, Kuwait, Malaysia, Thailand and Pakistan). The recent Hilsa production of Bangladesh is about half a million metric ton [4]. In spite of such importance Hilsa is still lacks molecular genomic information. Significance of this data for the improvement in sustainability and maintenance of diversity of this fish cannot therefore be overemphasized.

## Data description

Fresh *Tenualosa ilisha* samples from the river Padma at Rajshahi were collected and instantly preserved on dry ice. White and red muscles of the fish were used for DNA extraction. A modified SDS (Sodium Dodecyl Sulfate) method [5], optimized in our lab was used for DNA extraction (detailed methodology in Data File 1, Table 1).

**Table 1:** Overview of data files/data sets

Label	Name of data file/data set	File types (file extension)	Data repository and identifier (DOI or accession number)
Data file 1	DNA isolation and library preparation methodology	.docs file	https://figshare.com/s/467b8b670149f1a0617c
Data file 2	Whole genome assembly data	FASTA	NCBI GeneBank (Accession numbers: GCA_003651195.1) (http://identifiers.org/ncbi/insdc.gca:GCA_003651195.1.)
Data file 3	Whole genome sequence	FASTA	NCBI GeneBank (Accession numbers: QYSC01000001–QYSC01124209) (http://identifiers.org/ncbi/insdc:QYSC00000000.)
Data file 4	Annotation data file	.tsv	https://figshare.com/s/270b54d9d076ef5e5901

Pair end library with an insert size of around 300 bp was constructed for Illumina sequencing using NEB NebNext Ultra II DNA kit (detailed methodology in Data File 1, Table 1) Genomic DNA was sequenced by Illumina HiSeq 4000 and Pacific Bioscience Sequel, single molecule, real time (SMRT, Single Molecule Real Time) sequencing platforms. The quality of the reads were checked using FastQC [6]. MaSuRCA (Maryland Super-Read Celera Assembler) ver 3.2.6 was used for hybrid de novo assembly [7] using both the Illumina and PacBio data. The genome assembly data has been deposited in the NCBI GeneBank under the Accession numbers GCA_003651195.1 (Data file 2; Table 1). Illumina only data generated a fragmented assembly and showed 91% BUSCO [8] completeness. Addition of 15.7 Gbp data from PacBio significantly improved the quality and contiguity of the genome. Compared to Illumina only, N50 improved from 13 Kb (kilo base pair) to 188 Kb. Similarly, the total number of scaffolds reduced from 475,121 to 124,209. The assembled genome size of *Tenualosa ilisha* Padma Bangladesh is now 816 Mb (Mega base pair) and approximately 82% of the genome has been assembled. The BUSCO analysis revealing 95% completeness as well as significantly lower number of scaffolds and considerably better N50 indicates the genome to be of high-quality. The genome sequence data has been deposited in the NCBI GeneBank under the Accession numbers QYSC01000001-QYSC01124209 (Data file 3; Table 1). MAKER ver 3.0 pipeline [9] was used for structural annotation. GC content of the genome was determined to be 43.61%. RepeatMasker and Repeatmodeler using the latest version of repbase database [10–12] identified 27.27% repeat elements. Altogether, 31,254 gene models were predicted using the MAKER gene annotation pipeline based on both de novo and reference based predictions using genes/proteins from other fish species (Atlantic herring, carp, salmon, zebrafish). Out of the 31,254 genes, 24,648 were annotated using InterProScan [13] and 16,078 genes were found to have at least 1 GO (Gene Ontology) term assigned to them (Data file 4, Table 1). The Hilsa genome was found to be comparable to the Atlantic herring (807 Mb genome and 28,335 genes) [14] and to the genome of the common carp (1.8 Gb and 52,000 genes) [15].

## Limitations

The number of the regions unassembled in the genome is 4605 and the total number of bases positioned in this gap is 2,268,925 bp.

## Abbreviations

BUSCO: Benchmarking Universal Single-Copy Orthologs
PacBio: Pacific Bioscience
Gbp: giga base pair
Mb: mega base pair
Kb: kilo base pair
bp: base pair
GO: gene ontology
SDS: sodium dodecyl sulfate
EDTA: ethylenediaminetetraacetic acid
qPCR: quantitative polymerase chain reaction
SMRT: single molecule real time sequencing
MaSuRCA: Maryland Super-Read Celera Assembler
EST: expressed sequenced tag
SNAP: Semi-HMM-based Nucleic Acid Parser
